# Neuropsychological outcome in refractory obsessive–compulsive disorder treated with anterior capsulotomy including repeated surgery

**DOI:** 10.1111/pcn.13190

**Published:** 2021-01-20

**Authors:** Lenka Krámská, Dušan Urgošík, Roman Liščák, Lucia Hrešková, Jaroslava Skopová

**Affiliations:** ^1^ Department of Clinical Psychology Na Homolce Hospital Prague Czech Republic; ^2^ Department of Neurology Na Homolce Hospital Prague Czech Republic; ^3^ Charles University in Prague Prague Czech Republic; ^4^ Department of Stereotactic and Radiation Neurosurgery Na Homolce Hospital Prague Czech Republic; ^5^ Department of Psychiatry Na Homolce Hospital Prague Czech Republic

**Keywords:** anterior capsulotomy, cognitive tests, neuropsychology, stereotactic neurosurgery, treatment‐refractory obsessive–compulsive disorder

## Abstract

**Aim:**

Anterior capsulotomy (AC) is one of the last therapeutic options for obsessive–compulsive disorder (OCD) refractory to conservative treatments. Several forms of cognitive dysfunction have been identified after assessment of neuropsychological outcomes in OCD patients; however, few studies focused on cognitive changes in OCD patients after surgery. In the present study, we evaluated the effects of AC on cognitive performance and mood status in patients with refractory OCD.

**Methods:**

A total of 12 patients underwent bilateral AC between 2012 and 2019 at our institution. The patients (*n* = 12, female : male 5:7; mean age 39.7 years; duration ≥5 years) were assessed before and 6 months after intervention. The diagnosis of treatment‐refractory OCD was based on recommended criteria for surgical treatment. Patients were assessed using a neuropsychological battery and questionnaires focused on anxiety–depressive symptomatology. The Yale–Brown Obsessive Compulsive Scale was administered as a measure of severity of OCD symptoms.

**Results:**

We detected a significant decrease of OCD, and anxiety and depressive symptomatology assessed by Yale–Brown Obsessive Compulsive Scale, Beck Depression Inventory, and Beck Anxiety Inventory (*P* < 0.05) 6 months after AC in eight patients, and a partial decrease in four patients. Four patients underwent repeated AC with more pronounced improvement achieved after the first procedure. We did not detect decline in cognitive performance in any patients, but did find better visual memory performance (*P* < 0.05).

**Conclusion:**

AC reduced OCD and anxiety–depressive symptoms, and did not appear to influence cognitive performance, even after repeated surgery.

Obsessive–compulsive disorder (OCD) is a common, disabling, relapsing psychiatric illness. Combined cognitive behavioral therapy with pharmacotherapy is considered superior to either treatment as monotherapy, but is more expensive and several controlled studies^1^ have addressed the optimal treatment of OCD.^2^ As many as 10% of OCD patients do not respond to psychotherapeutic and pharmacological treatments as first‐line options and are therefore indicated to have refractory disease.^3^

Neurosurgical treatments are recommended for severely symptomatic patients without sustained response to cognitive behavior and pharmacological therapies.^4^ Anterior capsulotomy (AC) is considered a safe, well‐tolerated, and effective neurosurgical treatment method for refractory OCD,^5^ after which the majority of patients experience significant improvement. Lesions in the anterior limb of the internal capsule are introduced during the AC. They extend across the anterior thalamic projection fibers, and intervene with the fibers connecting the prefrontal cortex and mediodorsal thalamus. The underlying principle is supposed to be connected to the cortico‐striato‐thalamo‐cortical loop, which in OCD is assumed to be dysfunctional.^3^

Deep brain stimulation in the ventral capsule/ventral striatum and nucleus accumbens area is considered to be an effective therapy. The popularity of deep brain stimulation for OCD over ablative surgery is not caused by AC inefficacy, but likely because deep brain stimulation is perceived as more acceptable by patients and clinicians.^6^ Furthermore, patients living in distant areas are not suitable candidates for deep brain stimulation, as urgent stimulator programming issues or any complications could not be handled adequately and immediately. Therefore, bilateral AC still has a place in the neurosurgical treatment of psychiatric disorders, considering the efficacy and low cost of this procedure compared with deep brain stimulation.^7^

There are currently no definitive conclusions relating to neuropsychological findings after AC. Batistuzzo *et al*. reported that patients noted some level of improvement in cognitive functions, such as intelligence, verbal and visuospatial memory, and executive functioning.^8^

Several studies have been performed to evaluate and monitor neuropsychological disorders in OCD over an extended period of time. In recent studies, scientists have evaluated a wide range of cognitive domains, including memory (e.g. verbal, visual, and spatial memory) and executive function (set shifting, fluency, and decision making). The majority of these studies have shown impairments in visuospatial skills, visual memory, and visual and selective attention in OCD patients prior to surgery.^3^

Current functional magnetic resonance imaging (MRI) studies have shown correlations between neuropsychological impairments using neuropsychological tasks and clinical manifestations in OCD.^9^ However, as these studies lacked healthy controls, the type and degree of cognitive deficits before surgery, and the extent to which these deficits were alleviated by surgery, is still not clear. In addition, only a few studies have evaluated variations in cognitive functions at different time periods.^3^

Czigó *et al*. showed that both treatment options (i.e. AC and psycho‐ and pharmacotherapy) can be considered effective in reducing cognitive impairments, as well as OCD symptoms, however, to different levels.^10^ Neuropsychological and clinical improvements were stronger in the surgical group in comparison with the non‐operated group (treated by psycho‐ and pharmacotherapy). According to their conclusion, deficits in spatial working memory and attention are characteristic of patients refractory to the treatment.

In the present study, we investigated the cognitive and mood state before AC, and the effects of the procedure on cognitive performance, OCD symptoms, and mood status of patients with treatment‐refractory OCD 6 months after the stereotactic procedure.

## Methods

### Psychiatric evaluation

Patients were recommended from different outpatient psychiatric departments in the Czech Republic, just 12 of which fulfilled the inclusion criteria. OCD symptoms in our patients recommended for AC are presented in Table 1. The inclusion criteria for AC in our sample of OCD patients were based on recommended criteria for OCD surgical treatment: age 18–60 years, at least a 5‐year history of serious OCD symptoms reducing quality of life and activities of daily living, meeting the DSM‐IV or DSM‐V criteria of OCD diagnosis, Yale–Brown Obsessive Compulsive Scale (Y‐BOCS) score >25 and disease evaluated as treatment‐refractory by at least two psychiatrists, failure of both psychopharmacological treatment (3 SSRI or SNRI, 2 SSRI and clomipramine, 3 AP or AAP) and psychotherapy (cognitive behavioral therapy), OCD the primary diagnosis with very severe prognosis without surgical treatment, and satisfactory compliance. The presence of serious personality disorder, history of substance‐related and addictive disorders, central nervous system tumor, neurodegeneration or other organic CNS diseases, history of psychotic episode, and non‐compliance were considered exclusion criteria.

**Table 1:** OCD symptoms in patients recommended for AC

Patient	Dominant features
1.	Hand washing, disinfection, avoid touching door handles and different objects, time consuming hygiene rituals, floor and bathroom washing, avoiding cracks in sidewalk spots, etc.
2.	Hand washing, bathroom and toilet washing, avoid touching money and rubbish
3.	Checking door, car locks, windows, water taps, gas, ensuring not hurting someone
4.	Obsessional preoccupation with excrement in the street, ruminations of common events
5.	Checking door locks, gas, water, checking different spots, stops when walking to check did not lose something
6.	Checking door locks, gas, water, hand washing
7.	Hand and whole‐body hygiene rituals, laundering, avoid coat hangers due to potential contamination
8.	Checking e‐mail, words, numbers, hand washing, checking symmetricity of object positioning, keeping objects in a special position, arranging clothes with repetitive folding, time consuming kitchen cleansing before serving meals and cooking
9.	Different thoughts – how to handle them, ignore or not ignore them, questions – want to have and endure them, write or not write, etc.
10.	Obsession with contamination, infection esp. transmitted by birds, bats, checking apartment, looking for blood spots, checking food, checking seats in public transport, obsession with hurting, knocking someone down
11.	Obsession with violent scenes, acts, obscene images, obsession with obscene words when talking
12.	Obsession with contamination, doubts about knowledge acquired at school, revising, studying, persistent doubts about information correctness, looking for more and more sources

### Neuropsychological examination

Comprehensive neuropsychological assessment was performed in treatment‐refractory OCD patients who were treated by bilateral AC between 2012 and 2019. The patients (*n* = 12 female : male 5:7; mean age 39.7 years; duration ≥5 years) were assessed by a licensed clinical neuropsychologist (L.K.) before and 6 months after intervention at the Department of Clinical Psychology, Na Homolce Hospital, Prague, Czech Republic. Basic demographic characteristics are presented in Table 2. For the aim of this study, the patients were examined using a battery of neuropsychological tests and scales assessing anxiety–depressive symptomatology. The assessment was performed during one session and took 3–4 h and included the Wechsler Adult Intelligence Scale – short form, including subtests Block Design, Arithmetic, Similarities, Picture Completion; Digit Span; Digit Symbol; Rey Auditory Verbal Learning Test – immediate and delayed recall; Rey–Osterrieth Complex Figure Test (ROCFT) – copy, immediate, and delayed recall; Trail Making Test A/B; verbal fluency test – phonemic category. We also assessed anxiety–depressive symptomatology through the Czech version of the Beck Depression Inventory (BDI‐II); Beck Anxiety Inventory (BAI); and Montgomery–Åsberg Depression Rating Scale (MADRS). For evaluation of OCD manifestation The Y‐BOCS was used.

**Table 2:** Basic demographic variables

Demographic features	Mean (SD)
Sex (female/male)	5/7
Age at assessment (years)	39.7 (8.44)
Education (years)	15.5 (2.2)
Age at OCD onset (years)	18.17 (8.58)
Illness duration (years)	21.42

### Surgical technique of anterior capsulotomy

#### Imaging and target localization

The stereotactic procedure was performed with a Leksell Stereotactic system model G and planning software SurgiPlan (version Elekta Instruments, Stockholm, Sweden). MRI was performed on a Siemens Avanto 1.5 T scanner (Siemens, Munich, Germany), and included a 3D FLASH, T1‐weighted sequence (1.3 mm slice thickness), T2 SE, and PD‐weighted sequences (2 mm slice thickness). Intravenous contrast medium was applied to avoid intracranial vessels.

The anterior limbs of the internal capsule were chosen as the target for the ablative procedure in the vicinity of the anterior commissure with coordinates adjusted according to individual brain anatomy imaged by MRI: X = 11–19 mm from the midline; Y = 4–10 mm anterior to the anterior commissure; Z = 2–4 mm superior to the anterior–posterior commissure line.

#### Stereotactic surgery

**Fig. 1:**
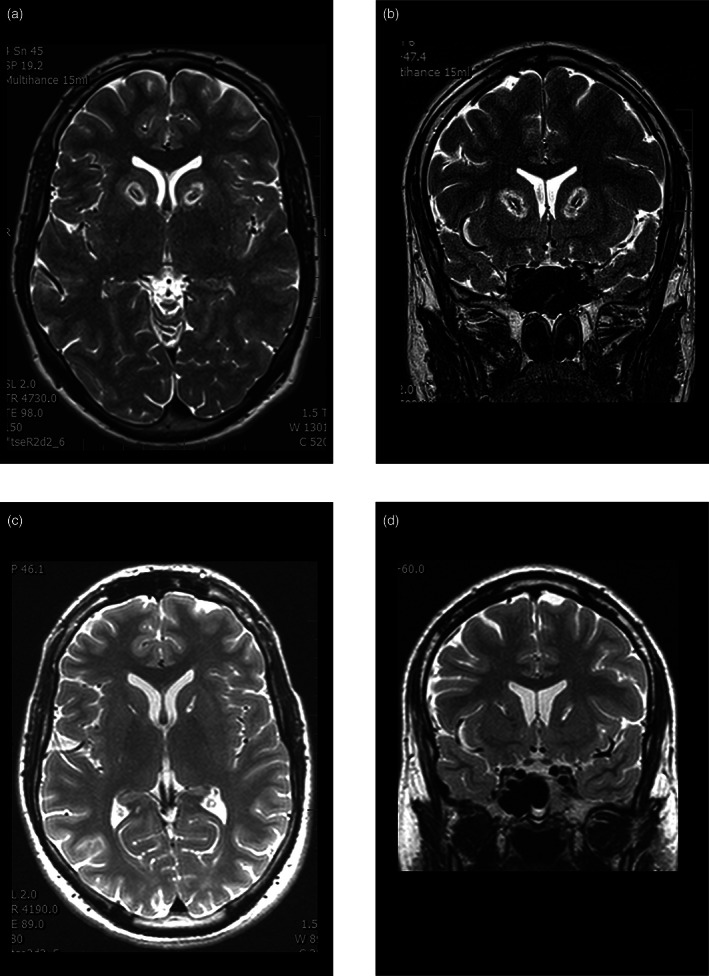
Magnetic resonance imaging examination, 1 day after thermolesion. Structural changes consisting of three coagulations in the right and left anterior limbs of the internal capsule. (a) Axial T2 SE image. (b) Coronal T2 SE image. Magnetic resonance imaging examination, 6 months after thermolesion. Reduced structural changes consisting of three coagulations in the right and left anterior limbs of the internal capsule. (c) Axial T2 SE image. (d) Coronal T2 SE image.

The procedure was carried out bilaterally under analgosedation and local anesthesia. A transdermal approach in the frontal area anterior to the coronal suture was performed using a 4‐mm drill. A unipolar electrode (16‐G; Diros Technology, Markham, ON, Canada) with a rounded end was gently and slowly inserted into the calculated target. The testing stimulation generated by the Diros neurostimulator at both high (100 Hz) and low (5 Hz) frequency was performed to verify the avoidance of eloquent structures. The first thermolesion was created by the same electrode and the same generator in the intended target. A further lesion was always shifted more frontal 5‐mm apart in the axis of the anterior limb of the internal capsule. In the first five cases, we performed just two lesions with a temperature of 75°C/60 s and 78°C/60 s bilaterally (four lesions in total). In the seven patients that followed, we performed three lesions bilaterally (6 lesions in total) with a temperature of 80°C/60s in the calculated target and 85°C/60 s in the upper positions of the electrode. The next day after surgery, we performed MRI with the same sequences, without administration of contrast medium, and assessment of the lesion localization and size was performed (Fig. 1)

#### Repeated surgery

In four patients, we enlarged the AC. The second procedure followed 6–19 months after the first surgery. The indication for the second operation was based on the evaluation of past events: (i) the patients subjectively assessed improvement of their condition as insufficient and wanted to undergo the second procedure; and (ii) improvement of Y‐BOCs score was at the lower limit of success rate among our patients. With one exception, a smaller‐volume lesion was introduced. Thus, just two lesions were performed bilaterally during the first procedure, contrary to other patients in whom we introduced three lesions during one operation. In three of these patients with two previous lesions, we only added two thermolesions just below and another above the previous lesions bilaterally (a total of four lesions were added). In one patient with three previous lesions, we performed new AC with three targets bilaterally, shifted 6‐mm anterior with respect to the last procedure. A temperature of 80°C/60 s was used.

### Psychometric measures

#### Global intellectual functioning

- The Wechsler Adult Intelligence Test 3rd revision short form was utilized to establish each patient's actual global intelligence performance based on Picture Completion, Digit Symbol, Similarities, Arithmetic, Digit span and Bloc Design subtests, and full‐scale intelligence. The Digit Span subtest is a valid and reliable measure of short‐term memory and auditory attention. Digit Span backwards measures working memory and mental manipulation. Block design measures the visuo‐constructional abilities.^11^

#### Language and verbal functions

Verbal functions were assessed using Czech versions of phonemic verbal fluency tests.

- The verbal fluency test (Controlled Oral Word Association Test) is a measure of phonemic verbal fluency, retrieval of words, and self‐regulation. Patients are told three letters of the alphabet. They are instructed to create words beginning with that letter within 1 min. The letters used in the Czech version were N, K, and P.^11^

#### Memory and learning (verbal and visual)

- The RAVLT assesses a wide range of functions: short‐term verbal memory, learning strategies, learning process, retention of information, and proactive and retroactive interference. The number of confabulation and confusion in memory processes, and differences between learning and retrieval are noted. A total of 15 unrelated words are repeated over five trials. A second list of 15 unrelated words is read to the patient and then the original list of words must be repeated. This is repeated after a 30‐min interval.^11^
- The ROCFT is a test used for the evaluation of visual memory and visuospatial constructional ability. The ROCFT includes three tasks: Copy, Immediate Recall, and Delayed Recall after a 30‐min interval. Recently, the ROCFT has proven useful for measuring executive function mediated by the prefrontal lobe.^11^

#### Attention and executive function

- Trail Making Tests A and B are tests used for evaluation of visual search and motor planning. Part A contains numbers, which have to be connected in ascending order (i.e. 1 to 2 to 3, etc.). Part B contains numbers and letters that must be connected, and patients alternate between these two (i.e. 1 to A to 2 to B, etc.). Part B is used to assess executive functions: patients have to inhibit the previous rule and shift between two items, while keeping the new rule in working memory.^11^

#### Emotional and mood status

- The Y‐BOCS measures types, severity, and amount of lifetime obsessions and compulsions. The Symptom Checklist (Y‐BOCS‐SC^12^) contains the most common OCD symptoms. Obsessions and compulsions are divided in 13 categories and two additional categories of miscellaneous symptoms.^13^ According to the study of Pallanti *et al*. a clinical response is considered an improvement of 25–35% from the baseline Y‐BOCS score, or a score of ‘much’ or ‘very much improved’ in the Clinical Global Impression of Improvement Scale.^14^ A total Y‐BOCS score of <16 (out of a total scale score of 40) means ‘remission’. ‘Relapse’ is defined as a worsening by 25% of the remission Y‐BOCS score (or a CGI‐I score of 6). ^14^
- The MADRS is a 10‐item rating scale that is used to assess the severity of depressive symptoms in patients with emotion and mood disorders. This scale includes questions on the following symptoms: apparent and reported sadness, tension, sleep and appetite disorders, attention and concentration deficits, lassitude, inability to feel, and pessimistic and suicidal thoughts.^2^ The total score ranges from 0 to 60, and higher MADRS scores indicate more severe depression.
- The BDI‐II is a 21‐item self‐reported questionnaire assessing the presence and severity of depressive symptoms. Each of the 21 items consist of 4‐point scale ranging from 0 to 3. A total score of 0–13 indicates minimal depression, 14–19 mild depression, 20–28 moderate depression, and 29–63 severe depression.^11^
- The BAI is a 21‐question self‐reported inventory that measures the severity of anxiety. Individual Likert scales range from 0 to 3, and total raw scores range from 0 to 63. Total score is classified as minimal (0–7), mild (8–15), moderate (16–25), or severe anxiety (30–63).^15^

### Statistical analysis

Values are reported as the mean and standard deviation (SD) for continuous variables, and as the number of participants (percent) for categorical variables. A paired *t*‐test was used to evaluate differences before and after the surgery. Differences in scores were evaluated by the Wilcoxon paired test. Statistical analyses were performed using Statistica vX (TIBCO Software Inc., Palo Alto, CA, USA). Changes in cognitive performance after the surgery were calculated by subtracting the baseline from 6‐month scores obtained in neuropsychological battery.

## Results

### Neurosurgical results

We did not observe any serious neurosurgical complications, such as intracranial bleeding, pneumocephalus, or infections. Patients were without new neurological signs. Postoperative MRI showed well‐localized thermolesions in the anterior limb of the internal capsule. The size of lesions was better represented on MRI performed a few months after AC, in comparison with examinations the first day after surgery (Fig. 1). The true extent of structural changes in the brain was actually much smaller than the first examination showed. These later examinations were carried out in seven patients.

### Clinical and psychiatric results after AC

After surgery, patients showed clinical symptomatic improvement 6 months postoperatively. A significant decrease in Y‐BOCS scores was observed in the majority of patients. Nevertheless, four patients considered the decrease of OCD symptoms as insufficient (Table 3) and they underwent a second AC. Two patients with an insufficient reduction in OCD symptoms (Y‐BOCS score reduction ≤35) were offered enlargement of AC, but they refused. The following results summarize the entire group, including the final condition of ‘insufficient’ patients after repeated stereotactic surgery. The Wilcoxon matched pairs test was used to detect the intervals at which these changes were statistically significant. At the 6‐month period, the mean Y‐BOCS score (16.58) was significantly lower than the mean score post‐surgery (31.67; *z* = 3.059, *P* = 0.002). As shown in Table 4, patients also showed a significant decrease in anxiety and depressive symptoms measured by MADRS, BDI‐II, and BAI (*P* < 0.05).

**Table 3:** Yale–Brown Obsessive Compulsive Scale scores before and after anterior capsulotomy

Patient	T0	T1	%	T2	%
1	36	23	36	23	36
2	29	8	72		
3	31	20	35	16	48
4	13	3	77		
5	28	24	14		
6	35	19	46		
7	34	17	50	16	53
8	36	20	44		
9	32	28	12		
10	40	10	75		
11	32	16	50	13	59
12	34	15	56		

T0, before anterior capsulotomy; T1, after anterior capsulotomy; T2, after repeated anterior capsulotomy.

**Table 4:** Neuropsychological performance and psychiatric status before and after anterior capsulotomy

Domain	Test	Preoperative mean (SD)	Postoperative mean (SD)	*P*‐value
Global intellectual Function	FS‐IQ	101.82 (11.17)	102.83 (11.13)	0.866
	WAIS‐III Picture Completion	18.09 (3.81)	19.25 (2.96)	0.563
	WAIS‐III Digit Symbol	47 (13.08)	53.25 (15.05)	0.075
	WAIS‐III Similarities	24.5 (5.14)	24.67 (4.48)	0.906
	WAIS‐III Arithmetic	14.36 (3.64)	15 (3.59)	0.759
	WAIS‐III Block Design	32.57 (13.84)	38.2 (12.66)	0.686
	WAIS‐III Digit Span	15.67 (4.44)	16.25 (4.27)	0.407
Verbal functions Executive function	Phonemic Fluency	37.58 (11.79)	36.75 (9.95)	0.754
Memory and learning	AVLT Trials 1–5 (Verbal)	45.75 (12.7)	49.83 (12.12)	0.053
	AVLT Long Delay Free Recall (Verbal)	8.33 (3.52)	8.92 (3.55)	0.386
	Rey Complex Figure Test – Immediate (Visual Spatial Memory)	15.86 (5.26)	20.04 (6.77)	**0.037**
	Rey Complex Figure Test – Delayed (Visual Spatial Memory)	16.05 (5.44)	20.29 (7.12)	**0.009**
Perceptual and Spatial function	Rey Complex Figure Test	32 (2.19)	32.17 (2.95)	0.859
Attention and executive function	Trail Making Test: Part A	40.5 (17.57)	38.25 (13.37)	0.308
	Trail Making Test: Part B	87.08 (40.54)	74.25 (43.62)	0.078
OCD symptomatology	Y‐BOCS	31.67 (6.73)	16.58 (7.44)	**0.002**
Emotional and mood status	BDI‐II	39 (12.22)	19.73 (16.58)	**0.008**
	MADRS	28.55 (9.09)	13 (9.93)	**0.003**
	BAI	31 (15.06)	18.08 (14.21)	**0.003**

Significant differences (*P* < 0.01) in both subgroups are highlighted in bold.

BAI, Beck Anxiety Inventory; BDI‐II, Beck Depression Inventory; FS‐IQ, Full‐Scale Intelligence Quotient; MADRS, Montgomery–Åsberg Depression Rating Scale; RAVLT, Rey Auditory Verbal Learning Test; WAIS‐III, Wechsler Adult Intelligence Scale 3rd Revision; Y‐BOCS, Yale–Brown Obsessive Compulsive Scale.

At a group level, we did not identify any deterioration in cognitive functions (Table 4). Six months after the surgery, we observed slight increases in cognitive performance in nearly all subtests on the cognitive battery. Significant improvements were detected in immediate and delayed visual memory (*P* < 0.05).

## Discussion

Cognitive behavioral therapy and pharmacotherapy are the first‐line treatment options conservatively used in the management and treatment of OCD. A subgroup of patients with OCD are refractory to these therapies and are considered as candidates for novel neurosurgical interventions.^16^ In the present prospective study, we compared the pre‐ and postoperative performance, and the safety and efficacy of AC used in severe forms of refractory OCD.

We detected a significant decrease in OCD, anxiety and depressive symptomatology assessed by the Y‐BOCS scale, BDI‐II, and BAI (*P* < 0.05) 6 months after AC in eight patients, and a partial decrease in four patients, in whom additional improvements were reached after repeated AC. Zhang *et al*. also reported that clinical symptoms (OCD, anxiety, and depression) evaluated by psychiatric scales were significantly decreased postoperatively.^17^ The recent clinical and research findings support the efficacy of AC in treating refractory OCD, and comorbid anxiety and depression symptoms.^18^ One of the most common comorbid disorders in patients with OCD is depression. When OCD symptoms of the primary diagnosis reduce, symptoms of comorbid depression and anxiety often alleviate as well.^19^

Our cohort was quite homogenous in manifestation of OCD. The majority of patients had checking, forbidden thoughts, or cleaning/contamination manifestations. Capsulotomy is considered as particularly ineffective for patients with dominant order and symmetry manifestations.^7^ Surgery is less effective in patients with hoarding manifestations too.^7^ Rück *et al*. also showed that effectivity of AC in patients with dominant order and symmetry manifestations is low.^20^

Few recent studies have systematically evaluated changes in cognitive functioning after AC. We did not detect any decline in cognitive performance measured by neuropsychological battery of tests and scales in our patients, even in those patients that underwent repeated surgery. We found better immediate and delayed visual memory performance (*P* < 0.05) at 6 months' follow up in our patients.

Currently, there is evidence from research studies that the clinical effectiveness of AC in patients with OCD is achieved with stable, and even improved cognitive functioning.^21^, ^22^ Batistuzzo *et al*. suggested that AC is not only a safe treatment option in terms of neuropsychological performance, but may improve particular cognitive functions, such as visuospatial memory.^8^ In a study by Gong *et al*., surgically‐treated OCD patients showed greater improvement in executive functions, verbal and visual memory, and visuospatial skills than patients without surgical intervention.^3^

The meta‐analysis of Shin *et al*. on OCD and neuropsychological assessment has noted that visuospatial memory has the most stable increase in OCD (visuospatial memory presented the largest effect size between all neuropsychological functions).^23^

The explanation for improved cognitive performance is still under debate. According to the study of Nyman and Andréewitch, neurosurgical interventions for psychiatric disorders can positively modify cognitive performance in various ways: improve cognitive functions due to the reduction of symptoms; and improve cognitive functioning by interruption of abnormal circuits.^24^ Disruption of hyperfunctional frontal–striatal circuits, which are involved in the pathophysiology of OCD, might indirectly or directly support restorative processes in the prefrontal cortex. It also positively influences visuospatial memory pathways by restoring dysfunctional visuospatial memory circuits.^8^, ^25^

Mantione *et al*. found no significant relationship between improved cognitive performance and clinical symptoms for treatment‐refractory OCD.^26^ Gong *et al*. concluded that cognitive domains, such as executive functions, probably require a longer restoration period.^3^ Zhang *et al*. found no differences in decision‐making between preoperative and short‐term postoperative evaluation.^17^ Decision‐making was improved to a level comparable to the healthy sample after a long period post‐surgery.

Several studies detected non‐verbal memory deficits in OCD preoperatively.^2^, ^3^, ^17^ Nakao *et al*. proposed that memory deficits in OCD are secondary to executive functions.^9^ The attention of patients focused on memorizing details negatively impact the whole learning process. According to current studies, miscellaneous views of memory impairments in OCD were described.^9^

Other clinical and anatomical adverse events, such as urinary incontinence, seizures, confusion, intracranial hemorrhage, cerebral edema, and others, are reported in the range of 0–66% in systematic reviews in cases of AC.^18^ However, we did not detect transient or permanent adverse events in our cohort. We used a gentle thermolesion procedure in collaboration with the conscious patient. The trajectories for electrode insertion were carefully selected, and magnetic resonance imaging with the help of a stereotactic planning station allowed us to avoid critical structures and larger vessels. Pepper *et al*. reported that, ‘serious adverse events are rare and often associated with specific surgical techniques (use of a leukotome^7^ or excessively high radiation doses with GK capsulotomy)', which was not the case in the present study.^27^

Long‐term follow ups of patients after AC are infrequent and potentially biased, because patients are evaluated and followed by the same treating specialist.^20^ We are convinced there must be strict inclusion criteria for patients considering potential complications and the irreversibility of this procedure. Our results support AC as a promising option for patients suffering from severe treatment refractory disorder.

In general, the present study reflects the Czech population, treatment options, and results of our area of interest. However, inconsistent findings of comorbid disorders in OCD across studies complicate generalization. Considering the significant impact of comorbidities, particularly depression, the quality of life is negatively influenced. Although the sample size of evaluated patients was relatively small, our findings may support future research regarding the suitability of AC for patients with refractory OCD.

In conclusion, the goal of the study was to evaluate changes of cognitive performance in OCD patients, OCD, and depressive–anxiety symptoms over a 6‐month postoperative follow‐up period. Our findings suggest that AC does not increase the risk of cognitive deterioration (even after repeated AC), on the contrary, we noticed significant improvement in visuospatial memory.

Anterior capsulotomy is a safe and effective therapeutic method for the treatment of refractory OCD in patients who have no other option to alleviate their symptoms and improve quality of life.^7^ We realize there are many sociopolitical and ethical problems and impacts related to ‘psychosurgery’. However, we are convinced that it is a reasonable treatment method used under critical and thoughtful regulation. Surgery remains the last solution, especially in a chronic intractable disorder and when conservative therapy has failed. AC requires careful multidisciplinary cooperation, and must not be performed without rigorous neuropsychiatric and neuropsychological testing.

## Disclosure statement

The authors declare no conflict of interest.

## Author contributions

L.K. and D.U. prepared the concept and design of the research study, and participated in the collection and analysis of data. L.K. wrote the original version of the manuscript and critically revised the manuscript; D.U., R.L., L.H., and J.S. participated in the collection of data, and critically revised and edited the manuscript.
